# Brain activity patterns induced by interrupting the cognitive processes with online advertising

**DOI:** 10.1007/s10339-017-0815-8

**Published:** 2017-06-12

**Authors:** Izabela Rejer, Jarosław Jankowski

**Affiliations:** Faculty of Computer Science and Information Technology, West Pomeranian University of Technology Szczecin, Żołnierska 49, 71-210 Szczecin, Poland

**Keywords:** Brain activity patterns, EEG, Online advertising, Interruptions, Cognitive processes

## Abstract

As a result of the increasing role of online advertising and strong competition among advertisers, intrusive techniques are commonly used to attract web users’ attention. Moreover, since marketing content is usually delivered to the target audience when they are performing typical online tasks, like searching for information or reading online content, its delivery interrupts the web user’s current cognitive process. The question posed by many researchers in the field of online advertising is: how should we measure the influence of interruption of cognitive processes on human behavior and emotional state? Much research has been conducted in this field; however, most of this research has focused on monitoring activity in the simulated environment, or processing declarative responses given by users in prepared questionnaires. In this paper, a more direct real-time approach is taken, and the effect of the interruption on a web user is analyzed directly by studying the activity of his brain. This paper presents the results of an experiment that was conducted to find the brain activity patterns associated with interruptions of the cognitive process by showing internet advertisements during a text-reading task. Three specific aspects were addressed in the experiment: individual patterns, the consistency of these patterns across trials, and the intra-subject correlation of the individual patterns. Two main effects were observed for most subjects: a drop in activity in the frontal and prefrontal cortical areas across all frequency bands, and significant changes in the frontal/prefrontal asymmetry index.

## Introduction

The role of digital media in marketing activities has grown from a niche strategy in the early nineties, to having a large share in today’s market (eMarketer 2012). Due to extensive research in many related fields, an evolution of the forms of advertising, from text and simple visualization to more complex digital content, is currently underway (Schultz and Patti 2009; Schibrowsky et al. 2007). Together with the growth of new possibilities, attention is being paid to techniques for both increasing the effectiveness and expanding the role of online advertising in marketing strategies (Neha et al. 2013). New techniques like behavioral targeting and social media marketing are being implemented, along with real-time optimization of marketing content on Web sites (Hauser et al. 2009). Growing effort is being put into creating engaging campaigns to attract customers’ attention, the results of which are analyzed with different techniques and factors affecting human behavior (Robinson et al. 2007).

The increasing presence of online marketing has fed the growth of a secondary market for tools to help users avoid most of it. This can be performed through specialized software for intentional avoidance (Krammer 2008; Krishnamurthy and Wills 2006), or mechanisms like banner blindness that minimize the level of user attention drawn by the non-editorial parts of Web sites (Benway and Lane 1998; Chatterjee 2008). To overcome the negative effects of such software and to acquire new customers, marketing companies apply different forms of advertising using more and more intrusive content (Edwards et al. 2002). Meanwhile, the level of intrusiveness has to be chosen very carefully because intrusive advertisements interrupt users’ cognitive processes and affect their perception of a brand (McCoy et al. 2007). Hence, as a result of overly intrusive online marketing, consumers may behave in the opposite way than expected and intended by the advertiser (Van Doorn and Hoekstra 2013). If a marketing campaign is to be effective, the level of online advertisement invasiveness has to be carefully balanced. To deal with this task, the influence of the advertisement on the user and his perception of the advertisement’s intrusiveness have to be measured with different dimensions. Research in this area uses concepts like subjective scales (Li et al. 2002); levels of perceived intrusiveness, irritation, informativeness, entertainment, and ad avoidance (Edwards et al. 2002); perceived loss of control (Azeem 2012); physical characteristics of advertising messages (Ha 1996); and combination of structural and functional approaches (Ha 2008; Rodgers and Thorson 2000)

One of the most often used approaches for measuring how a user perceives the intrusiveness of a marketing content was proposed by Li et al. (2002). According to this approach, advertisement intrusiveness is measured with a seven-point scale that defines the marketing content as: distracting, disturbing, forced, interfering, intrusive, invasive, and obtrusive. Each of the seven points of the scale can be described by a web user with one of seven levels, from “strongly agree” to “strongly disagree.” The approach proposed by Li et al. was used in different experiments by other authors (McCoy et al. 2007; Zha and Wu 2014).

Another method for measuring the intrusiveness of marketing content, based on forced exposure and psychological reactance, was used in a study conducted by Edwards et al. (2002). The assumption was that intrusive stimuli affect personal freedom and try to induce a change in user behavior, which inclines the consumer to attempt to restore his freedom of choice (Brehm and Brehm 1981). The editorial-ad congruence, interruption time, and cognitive intensity were changed among subjects. The levels of perceived intrusiveness, irritation, informativeness, entertainment, and ad avoidance were analyzed. The measure of intrusiveness was based on the seven-level scale proposed by Li et al. (2002).

In another study, carried out by Azeem, the consumers’ attitude toward spam and pop-up ads was analyzed according to three dimensions: ad interference, perceived loss of control based on psychological reactance, and the level of irritation (Azeem 2012). Ad interference was measured with the scale proposed by Li et al. (2002), irritation—with a seven-point semantic scale following the approach proposed by Fritz (1979), and perceived loss—with a scale from 1 (strongly disagree) to 7 (strongly agree) (Dowd et al. 1991). The research showed that users generally present a more negative attitude to spam than to pop-up advertising.

For subjective evaluation of advertisements, Ha (1996) used a method combining a structural approach with physical characteristics of advertising messages: quantity, intrusiveness, and competitiveness. The level of intrusiveness was evaluated by the degree to which an advertisement interrupted the flow of the main web content. Twelve years later, Ha and McCann (2008) proposed an integrated model for measuring intrusiveness, where information processing, structural, and functional approaches were connected.

Another interactive information processing model of Internet advertising merges functional and structural approaches with features affecting perceived intrusiveness, like vividness or movement (Rodgers and Thorson 2000). Rodgers and Thorson emphasize that, although the web content interrupting user’s workflow is perceived as frustrating, the user reaction depends on their motivation to explore the content. While goal-directed users react negatively only to advertisements that hinder their processing of required information, unfocused users find even less intensive advertising highly annoying.

Although most research on user attitudes toward marketing content relied solely upon subjective scales, some studies did incorporate quantitative attempts to measure intrusiveness. For example, Nielsen and Huber performed an experiment within a simulated online shopping environment in which the level of ad intrusiveness was related to the behavior of the closing button. The button worked immediately for less intrusive ads and with a delay for ads with higher intrusiveness (Nielsen and Huber 2009). The measure of real intrusiveness, i.e., the intrusiveness felt by the user, was the number of attempts to remove advertising content, showing the level of the user’s frustration. Kalyanaraman used a different approach based on the cognitive and social characteristics of interruptions (Kalyanaraman et al. 2005). The cognitive dimension was represented in his research by complexity, frequency, time of execution, and duration of interruptions, and the social dimension was represented by expectations and response to interruption. Yet another approach based on measuring the costs of expositions of annoying advertisements was proposed by Goldstein et al. (2013). During their study, two types of Web sites were compared with regard to the number of views: one containing dynamic annoying advertisements, and the other containing static ones. The outcome of the research was a theoretical model for computing dynamic equilibrium and the relation between user behavior with intrusive and non-intrusive content.

In summary, existing research has relied primarily upon subjective measures to evaluate web users’ attitude to marketing content. Prior experiments were usually conducted via online shops, and the users’ task was to directly evaluate the degree of intrusiveness with different subjective scales. Of course, some attempts have also been made to use more objective measures, such as the number of clicks (Nielsen and Huber 2009), costs of intrusiveness (Goldstein et al. 2013), and response time (Moe 2006), but the most objective approach—the direct analysis of a web user brain activity—has not yet been taken. More general EEG-based research on the influence of interruptions on brain activity has been carried out; however, none has focused on online advertising intrusiveness. As Beaton et al. state (2013), overall knowledge about brain activity during interruptions is very limited at this moment, and new research in this field is needed.

The aim of this paper is to report the results of the experiment, which was conducted to find the brain activity patterns associated with interruptions of the cognitive process by presenting internet advertisements during a text-reading task. The experiment was meant to serve as the beginning of a wider study, which aims to discover and analyze the changes in cortical activity patterns induced by different structures, layouts, and graphical patterns of internet advertisements presented during different cognitive processes. In order to achieve this broadly defined goal, first we had to determine whether the presentation of an internet advertisement while the subject was performing an intellectually demanding cognitive task would induce any changes in his/her cortical activity patterns. Hence, we designed an experiment during which a subject had to read a short text. While the subject was reading the text, the advertisements were displayed on the screen to disturb the subject’s cognitive process. The paper reports the results of this experiment with respect to the following questions:

1. Is it possible to find individual cortical patterns associated with an interruption of the cognitive process (text-reading task) by marketing content?
2. Are these individual cortical patterns stable across epochs?
3. If stable cortical patterns can be found for different subjects, is there any intra-subject correlation between individual patterns?

At the beginning of the experiment, we assumed that when the text-reading process was disturbed by the advertisement presentation, a negative emotion with approach motivation would be induced. Hence, we assumed that the cortical activity patterns corresponding to the interruption of the text-reading task should be consistent with one of the two competing anterior alpha asymmetry index theories. Originally, the anterior alpha asymmetry was referred to the valence dimension of emotion—the scientists claimed that the anterior alpha asymmetry index rises for positive emotions and drops for negative ones (Jones and Fox 1992; Canli et al. 1998). The primary positive–negative explanation of anterior alpha asymmetry is still popular among neuroscientists; however, there are also some works that do not support it, such as Lane et al. (1997); Pardo et al. (1993). One recent study, performed by Sourina and Liu (2011), revealed partial support for the positive–negative frontal asymmetry hypothesis. Although not all the subjects’ dominant hemispheres for positive and negative emotion were the same as expected in the asymmetric hypothesis, the pattern of lateralization for a particular subject was consistent among different experiments with a similar arousal level (Sourina and Liu 2011). Sourina and Liu concluded that the experiments may indicate that frontal lateralization exists with individual differences. For some individuals, the left hemisphere is indeed more active for positive and the right hemisphere is more active for negative emotions, but it could be the opposite for other individuals.

Another explanation of the anterior alpha asymmetry provides the approach–withdraw hypothesis. According to this hypothesis, the anterior alpha asymmetry is connected more with the experience of approach and withdraw motivation (a direction dimension of emotion) than with the valence dimension (Harmon and Allen 1998; Davidson 1992); the right–left anterior alpha asymmetry index increases for approach motivation and decreases for withdraw motivation.

Usually, positive emotions motivate toward the action and negative ones motivate to avoid the action. In such a case, both explanations of changes in the cortical activity index (the positive–negative explanation and the approach–withdraw explanation) agree with each other. However, when the positive emotion induces the withdraw motivation or the negative emotion induces the approach motivation, both theories provide inconsistent explanations. One such negative emotion with approach motivation is anger. Anger is an emotional response that appears when something blocks the process of obtaining an expected goal (Ekman and Friesen 1975). Anger automatically evokes tendencies toward aggression.

We believe that anger is the main emotion that is induced when the cognitive process is disturbed by advertisement presentation. Since anger motivates to take an action, we first expected that the cortical activity patterns established in the experiment would be consistent with patterns characteristic for approach motivation, which had been supported by the work of Harmon-Jones and Allen (1998). Hence, we expected an increase in the alpha asymmetry index. On the other hand, however, the emotion that was induced by the advertisement presentation could be a combination of anger alongside with irritation, frustration, or even depression. Some of these emotions also motivate to take an action, but others correlate instead with the withdraw motivation, which in turn corresponds to a drop in the alpha asymmetry index. These confounding variables rendered the experiment’s final output difficult to predict.

## Experiment setup

The experiment was performed with six subjects (five men and one woman), aged between 20 and 25. All subjects were right-handed without any history of mental disorders. Written informed consent was obtained from all subjects. The experiment was conducted according to the Helsinki Declaration on proper treatments of human subjects.

During the experiment, subjects were presented with a text, written in Polish (the native language for all participants). The task was to read the text, understand it, and answer 10 questions to test their understanding of it. The text was displayed on the computer screen in a web browser as 10 short pages, each of which contained about 300 words. The decision about when to display the next page was left to the subject (each page ended with a “next page” button). There was no option to return to previously read pages.

The text-reading process was interrupted by online advertisements. Ten advertisements were displayed, one per page. To avoid the habituation effect, the onset of each advertisement was chosen randomly between 5 and 15 s after a new text page was opened. All advertisements were displayed on the screen for the same amount of time (three seconds).

In order to draw the subject’s attention to the reading activity, the level of text understanding was evaluated at the end of the experiment with a questionnaire comprising 10 yes/no questions. Moreover, to make the subjects more agitated during the advertisements, the time needed to complete the whole experiment was measured. At the end of the experiment, the subjects were ranked and awarded according to the results achieved in both categories.

The detailed scheme of the experiment with one subject was as follows: The subject was placed in a comfortable chair, and the EEG electrodes were applied on his/her head. In order to limit the number of artifacts, the participant was instructed to stay relaxed and not move. When the subject was ready, the first part of the text was displayed on the screen and the EEG recording started simultaneously. The text was located in the middle part of the screen in a 5″ × 5″ dialog box. The dialog box featured a button for moving to the next page. The time allowed for reading a single text page was unlimited. As soon as the subject finished reading a page of text, he clicked the button and proceeded to the next page. The experiment ended when the subject clicked the button on the last text page.

Before the main experiment, the trial phase was carried out. In that phase, the subject was presented with a two-page text, designed in the same way as in the main experiment. The aim of the trial phase was to familiarize the subject with the task. During the trial phase, EEG data were not recorded.

**Fig. 1:**
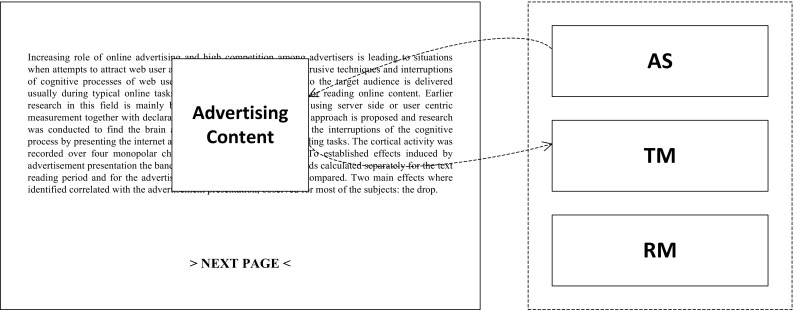
Structure of simulation environment: *AS* advertising server, *TM* tracking module, *RM* reporting module

The experiment was performed in an experimental web environment with an integrated text and advertising server displaying ads over the primary content of the Web site. The structure of the experimental environment is presented in Fig. 1. The presentation of the advertising content was controlled by the advertising server module (AS). Data related to web users’ activity were stored within a tracking module (TM), and final reports were generated by a reporting module (RM). The text was displayed within a web browser well known by the users (Firefox).

### EEG signal recording

The EEG data were recorded from four monopolar channels at a sampling frequency of 250.03 Hz. Six passive electrodes were used in the experiments. Four of them were attached to subject’ scalp at Fp1, Fp2, F3, and F4 positions according to the International 10–20 system (Jasper 1958). The reference and ground electrodes were placed at the right and left mastoid, respectively. The impedance of the electrodes was kept below 5 kΩ. The EEG signal was acquired with an EEG DigiTrack amplifier (Elmiko) and recorded with DigiTrack software.

### EEG signal preprocessing and epochs extraction

During the data recording stage, some restrictions to the experiment protocol were introduced in order to preliminarily limit the number of artifacts in the recording. However, despite these restrictions, some artifacts were still present in the signal. In order to eliminate them, filtering methods were applied. According to Fatourechi et al., low-pass filtering eliminates most EMG artifacts and high-pass filtering reduces EOG artifacts (Fatourechi et al. 2007). Hence, a simple band-pass filter (Butterworth filter of the fourth order, band 2–30 Hz) was used in the reported survey. By filtering out the activity outside the 2–30 Hz band, the power lines artifact (50 Hz—in Poland) was also eliminated.

Next, the epochs were extracted from the continuous signal recorded during the experiment. Each epoch started 3 s before the advertisement onset and ended when the advertisement was removed from the screen. Hence, the epoch lasted 6 s; during the first 3 s the subject was reading the text, and during the remaining 3 s he was looking at the advertisement. Since 10 advertisements were presented during the experiment, 10 epochs were extracted for each subject.

After extracting epochs, we inspected them visually in view of additional artifacts. On the assumption that the data analysis would be done on the basis of at least one second of continuous recording for both types of activities (text reading and advertisement watching), we looked for all epochs that contained at least one second of artifact-free continuous data recorded during the text-reading period and at least one second of artifact-free continuous data recorded during the advertisement presentation period. Visual inspection revealed that each epoch fulfilled our requirements, and hence, all 10 epochs for all six subjects were accepted for the analysis.

When the procedure described above was over, two periods for the analysis were chosen from each epoch, one for the text-reading activity, and the other for the advertisement-displaying part. The length of the period was variable and depended on the length of the artifact-free continuous recording. Hence, the length of the period varied from 1 to 3 s.

### Band power

As stated in introduction, we assumed that when we interrupt the text-reading process by presenting an advertisement, we induce a kind of negative (but approach) emotion in a subject. In most papers of EEG-based emotion recognition, the alpha, beta, and sometimes also theta frequency bands are reported as being the most discriminative for deciding on the actual emotional state of the subject. Usually, the signal power in each of these bands is analyzed (Aftanas et al. 2004; Liu et al. 2010), or the frontal asymmetry index is calculated (Harmon et al. 2004). Sometimes the beta/alpha prefrontal activity ratio is also used (Plass-Oude 2006).

In order to find the brain activity patterns related to the interruption of cognitive processes, we analyzed the signal power in 28 1-Hz frequency bands, starting at the 2–3 Hz band, and ending at the 29–30 Hz band. For each frequency band, channel, and epoch, we calculated two values—the average signal power in the period when a subject was reading a text (PT), and the average signal power in the period when an advertisement was displayed (PA). Moreover, in order to test the anterior hemisphere asymmetry, we calculated the anterior asymmetry index (i.e., *log right band power minus log left band power* (Davidson 1992)) for both pairs of corresponding channels: Fp2–Fp1 and F4–F3. The index was calculated not only for alpha sub-bands, but for all 28 1-Hz bands. For the sake of simplicity, in the further text the asymmetry indexes are referred to as two additional artificial channels, AI5 (the set of asymmetry indexes calculated for Fp2–Fp1) and AI6 (the set of asymmetry indexes calculated for F4–F3).

We also tested the beta/alpha activity ratio for Fp1 and Fp2 channels. We computed only three ratios per channel, using the classic frequency bands: alpha (8–13 Hz), low beta (13–15 Hz), beta (15–18 Hz), and high beta (18–30 Hz). Hence, the three beta/alpha indexes were: low beta/alpha (BA1), beta/alpha (BA2), and high beta/alpha (BA3).

### Statistical analysis

To find out whether there are any significant differences in the cortical activity recorded from a subject in both experimental conditions, we performed a statistical analysis of 10 results (10 advertisements) obtained for a subject. Since we wanted to know whether the interruption of the text-reading process brought a statistically significant difference in each frequency band and each channel separately, we performed 174 (28 frequency bands × [4 EEG channels + 2 asymmetry channels] + 3AB1 ratios + 3AB2 ratios) Student's *t*-tests instead of ANOVA. In the first 112 tests, we compared the average signal power in one frequency band and one channel calculated over 10 epochs for both conditions (with and without the advertisement). Hence, we tested the null hypothesis H_0_: *Average(PT*_*ch,f*_ *)* = *Average(PA*_*ch,f*_ *)* against the alternative hypothesis, H_1_: *Average(PT*_*ch,f*_) ≠ *Average(PA*_*ch,f*_ *)*, where *ch* channel index (*ch* = 1…4), and *f* frequency band index (*f* = 1…28). Next, we tested 56 hypotheses for asymmetry indexes computed for both conditions; H_0_: *Average(AI*_*ch,f*_ *)* = *Average(AI*_*ch,f*_ *)*; H_1_: *Average(AI*_*ch,f*_ *)* ≠ *Average(AI*_*ch,f*_ *)*, where *ch* channel index (*ch* = 5…6), and *f* frequency band index (*f* = 1…28). Finally, we compared the average values of three beta/alpha ratios calculated for both prefrontal channels; H_0_: *Average(BA*_*ch,in*_ *)* = *Average(BA*_*ch,in*_ *)*; H_1_: *Average(BA*_*ch,in*_ *)* ≠*Average(BA*_*ch,in*_ *)*, where *ch* channel index (*ch* = 1…2), and *in* ratio index (*in* = 1…3). All hypotheses were tested with paired-samples *t* test with 95% confidence level (*p* value = 0.05).

The further analysis was performed for all pairs of averages where the null hypothesis was rejected, i.e., for all pairs where both averages differed significantly. To find out the direction of the change induced by the advertisement presentation, we calculated the difference between the average value of signal power computed for the advertisement period and the average value of signal power computed for the text-reading period (*Average_power(advertisement)*-*Average_power(text)*).

Using the tests results, we established individual brain activity patterns for each of the six subjects. Next, we verified the stability of these patterns across succeeding epochs by counting the number of epochs consistent with the pattern. The analysis was performed individually for each subject. Finally, we compared the patterns established for different subjects to find out whether they had any common features.

## Results

Table 1 presents the direction of a change in the mean signal power or asymmetry index calculated over all 10 epochs individually per each subject. The “ + ” symbol means that the mean in the given frequency band and the given channel was greater when the advertisement was being presented; the “−” symbol means that the mean was greater when the subject was reading the text. Only significant results, tested with Student's *t*-tests (p-value 0.05), are presented in the table. To make the patterns more visible, the borders between four classic frequency bands (delta 0.5–4 Hz, theta 4–8 Hz, alpha 8–13 Hz, and beta 13–30 Hz) were highlighted.

**Table 1:** Direction of a change of the mean signal power (for channels: Fp1, Fp2, F3, and F4) or asymmetry index (for channels AI5, and AI6) calculated over 10 epochs individually per each subject

Subject	Frequency	Fp1	Fp2	F3	F4	AI5	AI6
**S1**	2–3 Hz				**+**	**+**	**+**
	3–4 Hz						**+**
	5–6 Hz	**−**				**+**	**+**
	6–7 Hz	**−**				**+**	
	7–8 Hz	**−**					**+**
	14–15 Hz		**−**		**−**		**−**
	18–19 Hz	**−**					
	21–22 Hz			**−**			
	24–25 Hz	**−**	**−**	**−**	**−**		
	29–30 Hz					**−**	
	BA1 [%]	**−**	**−**				
**S2**	9–10 Hz					**+**	
	10–11 Hz					**+**	
	11–12 Hz	**−**				**+**	
	16–17 Hz	**−**	**−**				
	20–21 Hz						**−**
	25–26 Hz						**−**
	26–27 Hz						**−**
	BA2 [%]		**−**				
**S3**	12–13 Hz				**−**		
	13–14 Hz	**−**		**−**			
	14–15 Hz			**−**			
	15–16 Hz			**−**			
	18–19 Hz					**+**	
	19–20 Hz			**−**			
	20–21 Hz					**+**	
	25–26 Hz				**−**		
**S4**	25–26 Hz						**+**
**S5**	10–11 Hz	**−**		**−**		**+**	**+**
	11–12 Hz			**−**			**+**
	13–14 Hz					**−**	
	17–18 Hz		**−**		**−**		
	19–20 Hz						**−**
	24–25 Hz		**−**				
	BA1 [%]	**+**					
**S6**	5–6 Hz					**+**	
	8–9 Hz						**−**
	15–16 Hz	**−**					
	BA2 [%]	**−**					

Plus “+” denotes the positive change between the text-reading period and the advertisement presentation period; minus “−” denotes the negative change. Only significant results, tested with Student’s *t*-test (*p* value = 0.05), are presented

**Fig. 2:**
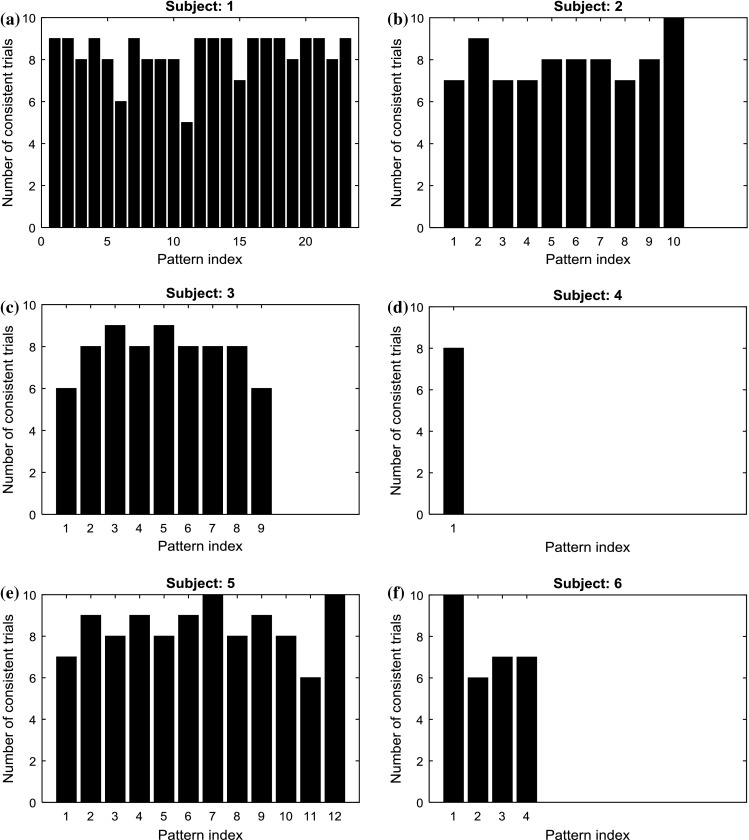
Patterns consistency across epochs for individual subjects. Succeeding patterns correspond to the patterns presented in Table 1. The detailed description of pattern indexes is given in the main text

In order to answer the second question posed in introduction, the question about the stability of the individual cortical patterns, a consistency analysis was performed (Fig. 2). Each of the six subfigures of Fig. 2 corresponds to one subject and presents his statistically significant cortical patterns. The horizontal axis of each subfigure denotes the pattern index, and the vertical axis—the number of epochs where the given pattern was found. The patterns were indexed according to rows of Table 1. Hence, for example the pattern no. 1 for S1 denotes the increase in the signal power in 2–3 Hz frequency band in F4 channel, and the pattern no. 3 for S1—the increase in the asymmetry indexes (AI5) in 2–3 Hz frequency band.

The full set of pattern indexes for succeeding subjects are given below.

- **S1** (Fig. 2a): 1: 2–3 Hz F4 +, 2: 2–3 Hz AI5 +, 3: 2–3 Hz AI6+, 4: 3–4 Hz AI6+; 5: 5–6 Hz Fp1−, 6: 5–6 Hz AI5+, 7: 5–6 Hz AI6+, 8: 6–7 Hz Fp1−, 9: 6–7 Hz AI5+, 10: 7–8 Hz Fp1−, 11: 7–8 Hz AI6 +; 12: 14–15 Hz Fp2−, 13: 14–15 Hz F4–, 14: 14–15 Hz AI6−, 15: 18–19 Hz Fp1−, 16: 21–22 Hz F3−, 17: 24–25 Hz F1−, 18: 24–25 Hz F2−, 19: 24–25 Hz F3−, 20: 24–25 Hz F4−, 21: 29–20 Hz AI5−, 22: BA1 Fp1−, 23: BA1 Fp2−;
- **S2** (Fig. 2b): 1: 9–10 Hz AI5 +, 2:10–11 Hz AI5 +, 3: 11–12 Hz Fp1–, 4: 11–12 Hz AI5+, 5: 16–17 Hz Fp1−, 6: 16–17 Hz Fp2−, 7: 20–21 Hz AI6−, 8: 25–26 Hz AI6−, 9: 26–27 Hz AI6−, 10: BA2 Fp2−;
- **S3** (Fig. 2c): 1: 12–13 Hz F4−, 2: 13–14 Hz Fp1−, 3: 13−14 Hz F3–, 4: 14–15 Hz F3–, 5: 15–16 Hz F3–, 6: 18–19 Hz AI5 +, 7: 19–20 Hz F3– 8: 20–21 Hz AI5 +, 9: 25–26 Hz F4–;
- **S4** (Fig. 2d): 25–26 Hz AI6 +;
- **S5** (Fig. 2e): 1: 10–11 Hz Fp1–, 2: 10–11 Hz F3–, 3: 10–11 Hz AI5 +, 4: 10–11 Hz AI6 +, 5: 11–12 Hz F3–, 6: 11–12 Hz AI6 +, 7: 13–14 Hz AI5–, 8: 17–18 Hz Fp2–, 9: 17–18 Hz F4–, 10: 19–20 Hz AI6–, 11: 24–25 Hz Fp2–, 12: BA1 Fp1 +;
- **S6** (Fig. 2f): 1: 5–6 Hz AI5 +, 2: 8–9 Hz AI6–, 3: 15–16 Hz Fp1–, 4: BA2: Fp1–.

The third question posed at the beginning of the experiment was related to an intra-subject correlation between individual patterns. In order to address that question, the statistically significant patterns established for individual subjects are gathered together in Table 2. The succeeding rows in Table 2 denote all frequency bands used in the survey (apart from those where no significant pattern was found for any subject), and the succeeding columns denote the increase and drop pattern for all six channels (four real and two artificial). The table cells contain the subjects’ indexes. The table is divided into three parts: the part containing information from low-frequency bands (from 2 to 13 Hz), the part containing information from higher-frequency bands (from 13 to 30 Hz), and the part containing information about the beta/alpha indexes.

**Table 2:** Patterns consistency across subjects

Band	Fp1		Fp2		F3		F4		AI5		AI6	
	+	**−**	+	**−**	+	**−**	+	**−**	+	**−**	+	**−**
2–3 Hz							1		1		1	
3–4 Hz											1	
5–6 Hz		1							1,6		1	
6–7 Hz		1							1			
7–8 Hz		1									1	
8–9 Hz												6
9–10 Hz									2			
10–11 Hz		5				5			2,5		5	
11–12 Hz		2				5			2		5	
12–13 Hz								3				
13–14 Hz		3				3				5		
14–15 Hz				1		3		1				1
15–16 Hz		6				3						
16–17 Hz		2		2								
17–18 Hz				5				5				
18–19 Hz		1							3			
19–20 Hz						3						5
20–21 Hz									3			2
21–22 Hz						1						
24–25 Hz		1		1,5		1		1				
25–26 Hz								3			4	2
26–27 Hz												2
29–30 Hz										1		
BA1 [%]	5	1		1								
BA2 [%]		6		2								

The numbers inside the cells denote indexes of subjects for whom the given pattern (described by table row and column) was found. Only statistically significant patterns are presented

## Discussion

### Individual patterns

The first part of this section discusses the patterns established for individual subjects. It should be reiterated that each pattern shows a drop or increase in the average signal power recorded from the given channel and filtered in the given frequency band during the advertisement presentation period. Hence, the “increase” pattern means that signal power was higher when the advertisement was being presented, and the “drop” pattern means that signal power was higher when the subject was reading the text.

**S1.** The major tendency that can be observed in the EEG signals recorded from the first subject is the increase in the asymmetry index in all sub-bands of the delta and theta bands. In some sub-bands, the increase appears in both the prefrontal and frontal areas (2–3 and 5–6 Hz), in others only in the prefrontal area (6–7 Hz), or in the frontal area (3–4 and 7–8 Hz). Because the asymmetry index calculated for the alpha band in an awake person is correlated inversely with cortical activity, a positive value denotes greater activation in the left hemisphere than the right (Davidson et al. 2000). Moreover, the alpha asymmetry index is negative for withdraw motivation and positive for approach motivation (Harmon and Allen 1998). The same relation has not yet been overtly confirmed for the delta and theta bands. However, as synchronic activity in the theta band can be detected when the subject is more relaxed (and the brain is more “inactive”) than when alpha oscillations are observed, we infer that the rules for the asymmetry index are also valid, at least for the theta band. Therefore, we believe that the positive asymmetry index calculated for the theta band indicates the approach motivation of the subject.

The drop in the right prefrontal and frontal activity was additionally confirmed by: the drop in the signal power in Fp1 in the 5–8 Hz band, the drop in the signal power in the low beta sub-band (14–15 Hz) in both right channels (Fp2 and F4), the drop in the low beta frontal asymmetry index (14–15 Hz), and the drop in the high beta prefrontal asymmetry index (29–30 Hz).

While the patterns found for lower-frequency bands and for the low beta band are entirely in agreement with the approach theory, the same cannot be stated about the patterns found in the middle and high beta bands. All patterns found in these bands are negative without any correlation with the hemispheres. In the 24–25 Hz band, a significant drop of the signal power was recorded even in all four frontal and prefrontal channels. This suggests that the advertisement’s interruption of the text-reading process lowered the subject’s overall concentration, which was reflected in the EEG recording as the drop of the signal power in beta sub-bands. The drop of both prefrontal activity indexes BA1 also confirms the overall drop of activity in this area.

**S2.** Almost all patterns obtained for subject S2 follow the approach theory. The positive alpha asymmetry index in the 9–12 Hz band, the negative beta asymmetry index in the 20–21 Hz and 25–27 Hz bands, the drop in alpha activity in the left prefrontal channel (Fp1, 11–12 Hz), the drop in beta activity in the right prefrontal channel (Fp2, 16–17), and the drop of the activity ratio (BA2) in the Fp2 channel all confirm the higher activity of the left hemisphere. The only exception is the drop in the signal power in the 16–17 Hz band in the Fp1 channel. Since this drop corresponds to the drop in the signal power in the Fp2 channel, it might be explained, as with subject 1, by the drop in the concentration level.

**S3.** Almost all statistically significant patterns established for subject S3 were found in the different sub-bands of the wide beta band. Only one pattern was located in the alpha band (12–13, F4). Most patterns found for this subject (apart from the negative pattern found for the F4 channel in the 25–26 Hz band) indicate the withdraw motivation. Two positive beta prefrontal asymmetry indexes (18–19 and 20–21 Hz), five negative patterns found for the Fp1 and F3 channels, and the drop in the alpha activity in the F4 channel (12–13 Hz) support the withdraw motivation theory. The only pattern that does not agree with the withdraw theory was the drop in activity in the F4 channel in the 25–26 Hz band. Unfortunately, without further research, nothing reasonable about the role of this pattern can be stated.

**S4.** Only one statistically significant pattern was found for subject S4—the positive high beta asymmetry index calculated for frontal channels. The positive value of the index indicates that during the advertisement presentation period the right frontal area was more active than the left one, which might be attributed to the withdraw motivation of the subject.

**S5.** All patterns found for subject S5 are consistent with each other. Moreover, since all of them underline the increase in activity of the left hemisphere or the drop in activity of the right hemisphere, all are in agreement with the approach theory. The positive alpha asymmetry indexes calculated for the 10–11 and 11–12 Hz bands correspond to the growing inactivity of the right hemisphere. The drop in activity in the 10–12 Hz band in the left frontal and prefrontal channels shows the growing activity of the left hemisphere. The drop in activity in the beta sub-bands (17–18 and 24–25 Hz) in the Fp2 and F4 channels, as well as the negative beta asymmetry index calculated for channels 13–14 and 19–20 Hz, is a result of the drop in right hemisphere activity. Finally, the positive value of the activity index (BA1) in Fp1 channel additionally confirms the growing activity of the left prefrontal hemisphere.

**S6.** Patterns found for subject S6 are not consistent with each other. The positive asymmetry index in the theta sub-band (5–6 Hz) indicates the higher inactivity of the right prefrontal hemisphere; however, the negative asymmetry index in the alpha sub-band (8–9 Hz) indicates higher inactivity of the left frontal hemisphere. Moreover, the drop in activity in the 15–16 Hz band in the Fp1 channel and the negative activity index (BA2) indicate a drop in activity in the left prefrontal hemisphere, which is not consistent with the positive value of the theta asymmetry index pointing to the higher activity of this area.

Since the patterns found for subject S6 were not consistent with each other, we decided to take into consideration the results from the pattern stability analysis. According to this analysis, the positive AI5 was found in all 10 epochs, the negative AI6 was found only in six epochs, and the two remaining patterns were found in seven epochs (Fig. 2f). Since the positive AI5 pattern seemed to be the most reliable one, we decided that the subject presents the approach motivation. Although we chose to describe the subject behavior using only one out of the four patterns, it does not mean that the remaining patterns are not important. All patterns chosen for the analysis were statistically significant, and hence, all of them should be regarded as important and should be taken into account in the analysis. However, some of them are difficult to explain at this stage of the survey without additional information. For example, the two negative patterns noted for the Fp1 band might have a connection with the drop in concentration level, but without further confirmation in the other channels this conclusion would be very uncertain.

### Patterns consistency for individual subjects

The general conclusion that can be drawn from the analysis of Fig. 2, presenting the stability of individual patterns found for each of the subjects, is that most of the patterns are highly consistent across the epochs. Only two out of all 65 patterns established for all the subjects appear in only 50% of epochs; five patterns appear in 60% of epochs, nine patterns appear in 70% of epochs, and the remaining 49 patterns appear in 80–100% of epochs. Since more than 75% of the patterns appear in at least 80% of epochs, it can be said that the patterns established for the individual subjects are stable.

What is even more important when talking about the patterns’ consistency is that the patterns found for the majority of subjects were consistent with each other. To be specific:

- all patterns found for subject S5 were in agreement with the approach theory;
- all patterns but one found for subject S2 were in agreement with the approach theory;
- all patterns but one found for subject S3 were in agreement with the withdraw theory;
- the only pattern found for subject S4 was in agreement with the withdraw theory;
- more than 60% of patterns found for subject S2 were in agreement with the approach theory, and the remaining patterns consequently showed a drop in the concentration level.

Only the patterns found for subject S6 were not consistent with each other or with the current theories explaining activity in the frontal and prefrontal cortical areas.

### Patterns Consistency across the Subjects

The patterns were highly consistent for individual subjects, but at the same time they differed among the subjects. The pure analyses of the descriptions of the individual patterns did not reveal any similarity between subjects. However, when the individual patterns were gathered together in one table (Table 2), some interesting facts could be noted.

1. In general, the negative activity patterns could be observed in frontal and prefrontal channels in almost all frequency bands (there was only one exception to that rule: the positive pattern in the 2–3 Hz band in the F4 channel for subject S1). Since the activity decreased so in the alpha as in the beta sub-bands, it could mean that the advertisement presentation induced two effects: first, the drop in concentration level (attributed to the decreasing activity in the beta sub-bands) and second, the drop in relaxation level (attributed to the decreasing activity in the lower-frequency bands). Both effects are very probable because the abrupt interruption of the reading task had to disturb the subject’s concentration on the task and at the same time had to make him feel uneasy.
2. The asymmetry index calculated for the lower-frequency bands (from delta to alpha, 2–13 Hz) was positive for both frontal cortex and prefrontal cortex. Also, this rule had one exception—the AI6 index for subject S6 was negative in the 8–9 Hz band. It should be noted, however, that the stability of this inconsistent pattern was relatively small, as the pattern appeared in only six out of 10 epochs (Fig. 2f). The positive frontal and prefrontal asymmetry index rule was true for four out of six subjects, those for whom the approach theory seemed to be valid.
3. Also, the asymmetry index calculated for higher-frequency bands (from low beta to high beta, 13–30 Hz) differed significantly for both analyzing conditions. However, the direction of the changes in this index was not consistent across the subjects. Hence, further research is needed to explain the different behaviors of the asymmetry index in higher-frequency bands.
4. Referring to the activity indexes BA1 and BA2, it can be said that for three out of six subjects (S1, S2, and S6), at least one index decreased when the advertisement was presented. This additionally supports the hypothesis about the overall drop in activity in the prefrontal area. At first glance, the opposite conclusion should be drawn for subject S5, for whom the left BA1 index was positive. However, when the approach motivation of this subject is taken into consideration, such a conclusion is not so straightforward, since it is not known whether the increasing activity related to the approach motivation covered the decreasing activity related to the drop in concentration. The activity indexes calculated for two other subjects (S3 and S4) did not differ significantly for both experimental conditions.

## Conclusions

The research that has been conducted so far to discover how intrusive marketing affects internet users has typically focused on analyzing the impact of online advertisements on brand awareness and memory (Nielsen and Huber 2009). The surveys were based mostly on analyzing the directions of the users’ visual attention, on recording their behavior during field or controlled experiments, and on analyzing their answers given in questionnaires, provided both on real Web sites and in test environments (Moe 2006; Nielsen and Huber 2009). In the presented paper, we proposed another strategy for investigating the influence of intrusive advertising on a user’s behavior and emotional state. We proposed to measure this influence by means of the most direct and objective approach—by recording the EEG signals from the user’s brain.

Two main conclusions about the brain activity of the subjects participated in the experiment can be drawn from the patterns presented in the paper. First, the advertisement presentation decreases the subject’s concentration and so induces an overall drop in beta activity in the frontal/prefrontal regions. Second, the advertisement presentation induces changes in the frontal/prefrontal asymmetry index. The direction of this change differs, however, among the subjects.

Our hypothesis is that the subject response for the advertisement might depend on the subject motivation predisposition. If the subject is more approach oriented, the changes in the asymmetry index might reflect the growing activity of the left hemisphere. If, on the other hand, the subject is more withdraw oriented, these changes might reflect the growing activity of the right hemisphere. This, however, is only a hypothesis, which has not been tested yet. It seems that to verify this hypothesis, first the overall motivation direction of the subject should be established. This can be a main direction of our future work together with testing different forms of online advertisements and levels of intrusiveness.
